# Optimization problem of bike-sharing system rebalancing based on dynamic pricing

**DOI:** 10.1371/journal.pone.0347218

**Published:** 2026-04-16

**Authors:** Jianrong Cai, Qiang Wen, Tao Chen, Lian Xie, Jie Yu, Yang Liu

**Affiliations:** 1 School of Civil Engineering, Hunan City University, Yiyang, China; 2 School of Transportation, Changsha University of Science & Technology, Changsha, China; 3 School of Traffic and Transportation, Lanzhou Jiaotong University, Lanzhou, China; Hamad Bin Khalifa University College of Science and Engineering, QATAR

## Abstract

Bike-Sharing Systems (BSS) help address first- and last-mile travel challenges. As a limited public resource, the current pricing mechanism fails to fully exploit the service potential of bike-sharing or adequately capture heterogeneous user price sensitivity. We propose a Dynamic pricing-based Stochastic Demand-Response Optimization (DSDRO) method to address this gap. The method balances service fairness under varying demand stickiness while maximizing enterprise resource utilization. The DSDRO framework leverages spatiotemporal patterns in bike-sharing demand data and introduces a dynamic pricing model that differentiates user demand stickiness. This model is embedded in a two-stage allocation-rebalancing formulation. An improved Particle Swarm Optimization algorithm enhanced with Large-scale Neighborhood Search (PSO-LNS) is designed to solve the model, yielding the optimal bike allocation and dispatch routes at each node. Numerical experiments based on real operational data validate the proposed approach. Compared to a traditional genetic algorithm baseline, DSDRO increases expected revenue by 15.51% and net profit by 24.18% under identical resource conditions. An ablation study shows that dynamic pricing alone increases revenue by over 300% relative to fixed pricing, while the LNS component reduces rebalancing cost by 76.21% relative to pure PSO. Algorithm stability is confirmed through 10 independent runs, with all performance metrics exhibiting a coefficient of variation below 5%. These results suggest that DSDRO shows promise in improving the utilization of bike-sharing resources, though further validation across diverse operational contexts is needed before broader conclusions can be drawn.

## 1. Introduction

With the rapid development of bike-sharing systems (BSS), growing attention has been paid to their significant potential in urban transportation. As indicated by the Bike-sharing World Map, since 2023, over 2,000 BSS have been implemented in 92 countries globally, with approximately 80 more under construction. As a green and convenient mode of transportation, scholars are optimistic about the significant role of BSS in alleviating urban traffic congestion and reducing environmental impact [1,2]. However, the sustainability of BSS operations remains a critical concern: nearly 1,900 systems have closed or suspended operations due to poor management and low profitability(https://bikesharingworldmap.com), forcing them out of the market. Even well-established enterprises like Mobike and Velib must operate cautiously and strategically [3]. Therefore, improving operational quality to maintain stable profitability is essential for the long-term, healthy development of BSS.

A central challenge facing BSS operators is the spatial and temporal mismatch between bike supply and user demand, commonly addressed through rebalancing strategies. The existing literature on rebalancing allocation schemes is extensive, spanning dynamic adjustment and static distribution approaches. Dynamic adjustment methods leverage machine learning and data mining techniques to rebalance bikes in real time based on user behavior [4–6], while static distribution approaches focus on station capacity and demand forecasting to pre-allocate bikes and improve overall system utilization [7–9]. In addition, some studies have provided comprehensive reviews of rebalancing patterns, strategies, and influencing factors at the system level [10,11], and others have incorporated user satisfaction into resource allocation decision frameworks to enhance service quality [12]. More specifically, rebalancing strategies can be categorized as supply-demand-based [13], user behavior-based [14,15], and geographic information-based [16,17]. However, most existing studies focus primarily on minimizing rebalancing costs—such as total travel distance, scheduling time, and operating expenses—while neglecting the direct impact of operational decisions on corporate revenue. In practice, cost reduction alone cannot guarantee the long-term viability of BSS enterprises; only by ensuring stable profitability can operators continue to provide high-quality services to society.

Beyond rebalancing, pricing strategy is a crucial yet underexplored lever for improving BSS operational performance. Dynamic pricing—adjusting fees based on demand fluctuations and market conditions—has demonstrated considerable promise in balancing supply and demand while maximizing revenue. Existing dynamic pricing research primarily pursues two objectives: maximizing total system profit or social welfare at the macro level [18,19], and improving system utilization and spatial balance [20–22]. Some studies have analyzed travel pattern differences between docked and dockless bike-sharing users, revealing the heterogeneity in price responsiveness across user groups and providing empirical foundations for differentiated pricing [23]. Others have explored the integration of green constraints and route optimization into scheduling frameworks, offering new perspectives for the coordination of pricing and dispatch [24]. A number of studies model dynamic pricing as sequential decision-making problems, addressed via model predictive control [25], dynamic programming [26], and reinforcement learning [27], respectively. Another stream of research employs pricing as an incentive mechanism to encourage users to participate voluntarily in rebalancing, thereby reducing operational costs [28,29]. However, these incentive-based approaches do not fundamentally address pricing from the enterprise’s perspective; they treat users as auxiliary rebalancing agents rather than as demand segments with heterogeneous price sensitivity. Critically, existing pricing models largely apply uniform or undifferentiated charges, failing to distinguish users’ demand stickiness—the degree to which different users depend on bike-sharing and their tolerance for price variation. This limitation results in less-dependent users occupying shared resources that could otherwise serve more committed users, reducing the overall efficiency and fairness of resource utilization.

The interplay between pricing and rebalancing further highlights a structural gap in the literature: allocation schemes, rebalancing plans, and charging measures are most often studied in isolation, yet in practice they jointly determine enterprise net profit. An allocation scheme directly affects user satisfaction and vehicle utilization; a rebalancing strategy shapes operational costs through routing efficiency; and a pricing mechanism links user demand to resource availability. Optimizing any single dimension independently cannot guarantee global optimality or long-term profitability. What is urgently needed is an integrated framework that jointly considers dynamic pricing, resource allocation, and rebalancing scheduling, driven by enterprise revenue maximization rather than cost minimization alone.

To fill this gap, this paper proposes a Dynamic pricing-based Stochastic Demand-Response Optimization (DSDRO) method. The main contributions are as follows:

(1) A demand-differentiated dynamic pricing model is established by incorporating departure time, riding distance, and node importance. The model differentiates users’ demand stickiness by charging higher fees during peak periods while maintaining accessibility for price-sensitive users, thereby improving the fairness and efficiency of bike-sharing resource utilization.
(2) A two-stage joint optimization model targeting enterprise net profit is constructed. The first-stage allocation model determines the optimal bike deployment at each node under dynamic pricing; its output directly feeds into the second-stage rebalancing model, which optimizes dispatch routes to minimize operational costs. This integrated structure jointly optimizes pricing and routing decisions within a unified profit-maximization framework.
(3) An improved Particle Swarm Optimization algorithm enhanced with Large-scale Neighborhood Search (PSO-LNS) is designed to solve the joint model, providing enterprises with revenue-oriented dispatch plans, with the potential to inform operational practice in broader real-world settings pending further validation.

The remainder of this paper is organized as follows. Section 2 presents the DSDRO model, including the dynamic pricing mechanism and the joint allocation-rebalancing optimization formulation. Section 3 describes the PSO-LNS algorithm designed to solve the model. Section 4 reports numerical experiments and comparative analyses. The Conclusion section summarizes the findings, discusses managerial implications, and outlines directions for future research.

## 2. Allocation and rebalancing model based on dynamic pricing

### 2.1. Problem description and parameter definition

Bike-sharing enterprises always aim to maximize the net profit of the system while controlling dispatch costs by formulating reasonable dispatch strategies. Therefore, the problem of maximizing the net profit of bike-sharing enterprises can be transformed into: how to reasonably plan the number of bike-sharing and dispatch routes at each node in the area, to maximize the difference between the expected revenue of bike-sharing and the dispatch cost.

To establish a reasonable model, the following assumptions are made under full consideration of the actual situation:

(1) It is assumed that each area has only one dispatch center, from which all dispatch trucks depart and to which they return after dispatching;
(2) Each dispatch truck can visit the same node only once, but the same node can be visited by multiple dispatch trucks;
(3) The coordinates of the dispatch center and nodes are fixed and unchangeable;
(4) Special dispatch factors such as special weather or traffic conditions are not considered;
(5) Damage to and recovery of bike-sharing are not considered;
(6) The dispatch trucks travel at the maximum safe average speed during the dispatch process.
(7) Users’ demand for bike-sharing is sensitive to pricing, and the demand response to price changes can be characterized by a price elasticity coefficient.

The main notations and parameter definitions used in this paper are detailed in Table 1.

**Table 1:** Model parameters.

	Parameter Symbols	Parameter Meanings
	K=(1,...,k,...,s)	Truck set
Parameters	*O*_*i*_	Number of bike-sharing at node *i* before dispatch
	*S*_*i*_	Number of bike-sharing at node *i* after dispatch
	*E*_*i*_	Expected number of bike-sharing at node *i*
	*V*	Maximum loading capacity of the dispatch truck
	α	Penalty coefficient for under-supply deviation from expected demand (α>β)
	β	Penalty coefficient for over-supply deviation from expected demand
	*D*	Maximum driving distance of a dispatch truck
	*F*	Fixed cost of a dispatch truck
	$c_{\text{0}}$	Basic riding fee of bike-sharing
	ε	Price elasticity coefficient of demand
	*u*	Unit distance dispatch cost of a dispatch truck
	$Q_{i,j}^{k}$	Number of bike-sharing on dispatch truck *k*, calculated per node
	*M*	A very large positive number
	$w_{ij}$	Directional weight from node i to node j
	$P_{ij}$	Transition probability from node i to node j
	$q_{i}$	Number of bike-sharing deployed at node i
	$\mu_{t}$, $\sigma_{t}$	Mean and standard deviation of departure time distribution
	$D_{i}^{\text{0}}$	Baseline demand at node i under standard pricing
Decision Variables	$\delta_{k}$	If dispatch truck k is deployed, then 1; otherwise 0
	$x_{ij}^{k}$	0-1 variable. If dispatch truck *k* passes through dispatch arc (*i*, *j*), then it is 1, otherwise 0
	$y_{i}^{k}$	Number of vehicles loaded and unloaded by the *k*th truck at nodes *i* and *j*. For loading at node *i*, it is $y_{i}^{k}\geq 0$; for unloading at node *j*, it is $y_{j}^{k}\leq 0$

### 2.2. Pricing model based on dynamic charges

The demand for bike-sharing is not entirely random. According to the analysis of bike-sharing order data, the usage of bike-sharing generally follows a normal distribution, reaching a peak in certain periods. During peak periods, the demand for bike-sharing is high while the supply is limited. In such situations, enterprises can guide user usage during peak periods by adjusting fees, thereby improving enterprise revenue and meeting user needs. Hence, this paper proposes a dynamic pricing method for bike-sharing operations. The empirical data used in this study are sourced from the 2021 Digital China Innovation Contest; a comprehensive description of the dataset, including preprocessing procedures and associated limitations, can be found in [30].

Bike-sharing stations play different roles across nodes. Based on spatial analysis of bike-sharing order data, we adopt node demand as a proxy for node importance. The greater the demand of a node, the higher its importance. We first rank the predicted demand values of all *n* nodes in ascending order. Let $w_{i}$ denote the rank of node *i* among all nodes in terms of demand importance. To reflect the asymmetry in riding importance—riding toward a high-demand node should be weighted higher than riding away from it—we define the directional weight from node i to node j as:


$$w_{ij}=\frac{w_{j}}{max\left(w_{i},w_{j}\right)}$$ (1)

This ensures that $w_{ij}\in \left(0,1\right)$, with $w_{ij}=1$ when the destination node j is at least as important as the origin node i, and $w_{ij}<1$ when the destination is less important. This asymmetric design reflects the practical consideration that rides toward high-demand nodes generate more system value. Accordingly, the weight matrix W from node i to node j can be obtained:


$$W=\left[\begin{array}{cccc} w_{11} & w_{12} & \cdots & w_{1n}\\ w_{21} & w_{22} & \cdots & w_{2n}\\ \vdots & \vdots & \ddots & \vdots \\ w_{n1} & w_{n2} & \cdots & w_{nn} \end{array}\right]$$ (2)

The distance traveled by the user from the origin node to the destination node is considered as the user’s basic riding cost. We assume that the user always chooses the shortest riding route from node i to node j. The riding distance can be determined based on the coordinates of the electronic fences at each node. The distance matrix can be obtained:


$$d_{ij}=\left[\begin{array}{cccc} d_{11} & d_{12} & \cdots & d_{1n}\\ d_{21} & d_{22} & \cdots & d_{2n}\\ \vdots & \vdots & \ddots & \vdots \\ d_{n1} & d_{n2} & \cdots & d_{nn} \end{array}\right]$$ (3)

During peak periods, the cost of riding varies with different departure times. According to the analysis of departure times, the usage of bike-sharing overall shows a normal distribution. This paper combines dynamic pricing with the normal distribution characteristics of bike-sharing demand.

Let τ(t) denote the departure rate of bike-sharing at time t, which follows a normal distribution:


$$\tau \left(t\right)=\frac{\text{1}}{\sqrt{2\pi }\sigma_{t}}e^{-\frac{(t-\mu_{t}{)}^{\text{2}}}{2\sigma_{t}^{\text{2}}}}$$ (4)

where $\mu_{t}$ and $\sigma_{t}$ are the mean and standard deviation obtained from fitting historical order data.

Since τ(t) is a probability density function with dimension $time^{-1}$, it cannot be directly used as a dimensionless pricing weight. We define the departure time pricing weight as:


$$\psi \left(t\right)=1+\alpha_{p}\cdot e^{-\frac{(t-\mu_{t}{)}^{\text{2}}}{2\sigma_{t}^{\text{2}}}}$$ (5)

where $\alpha_{p}>0$ is the peak pricing premium coefficient. This ensures $\psi \left(t\right)\in [1,1+\alpha_{p}]$, with $\psi \left(\mu_{t}\right)=1+\alpha$ at the peak time $t=\mu_{t}$ (maximum surcharge above the base rate) and ψ(t)→1 for off-peak times (standard rate). A larger $\alpha_{p}$ results in a stronger peak surcharge, more effectively discouraging peak-period usage. A smaller $\sigma_{t}$ results in a sharper pricing peak, concentrating the surcharge around peak hours; a larger $\sigma_{t}$ spreads the pricing effect over a wider time range, encouraging users to shift departures over a longer period.

To characterize the flow pattern of bike-sharing between nodes, we estimate the transition probability from node i to node j directly from historical OD (Origin-Destination) data. Let $N_{ij}$ denote the observed number of trips from node i to node j in the historical data, then:


$$P_{ij}=\frac{N_{ij}}{\sum_{j=1}^{n}N_{ij}}$$ (6)

This ensures $\sum_{j=1}^{n}P_{ij}=1$ for each origin node i. The transition probability matrix P is:


$$P=\left[\begin{array}{cccc} p_{11} & p_{12} & \cdots & p_{1n}\\ p_{21} & p_{22} & \cdots & p_{2n}\\ \vdots & \vdots & \ddots & \vdots \\ p_{n1} & p_{n2} & \cdots & p_{nn} \end{array}\right]$$ (7)

Remark. If historical OD data is insufficient, the transition probability can be approximated by:


$$P_{ij}\approx b_{i}\cdot r_{j}=\frac{D_{i}}{\sum_{i=1}^{n}D_{i}}\cdot \frac{S_{j}}{\sum_{j=1}^{n}S_{i}}$$

where $D_{i}$ is the borrowing demand at node i and $S_{j}$ is the returning demand at node j. However, this independence approximation should be validated against actual OD patterns, as it ignores spatial correlations between origins and destinations.

Based on the above factors, the riding fee charged to a user departing at time t from node i to node j is defined as:


$$f_{ij}\left(t\right)=w_{ij}\cdot d_{ij}\cdot \psi \left(t\right)\cdot c_{\text{0}}$$ (8)

where $c_{\text{0}}$ is the basic riding fee per unit distance. This formula reflects that the riding fee is proportional to distance, weighted by the directional importance of the route and the departure time.

Dynamic pricing affects user demand. We introduce a price elasticity mechanism to capture this feedback. Let $D_{i}^{\text{0}}$ denote the baseline demand (under standard pricing) at node i, and ${\overset{―}{f}}_{i}$ denote the average riding fee at node i under dynamic pricing. The adjusted demand at node i is:


$$D_{i}\left({\overset{―}{f}}_{i}\right)=D_{i}^{\text{0}}\cdot {\left(\frac{{\overset{―}{f}}_{i}}{f_{\text{0}}}\right)}^{-\varepsilon }$$ (9)

where $f_{\text{0}}$ is the reference (standard) fee and ε>0 is the price elasticity coefficient. When ε is large, demand is highly sensitive to price changes; when ε is small, demand is relatively inelastic.

The time-averaged riding fee from node i to node j, weighted by the departure rate, is defined as:


$${\overset{―}{f}}_{ij}=\frac{\int_{t_{s}}^{t_{e}}f_{ij}\left(t\right)\cdot \tau \left(t\right)dt}{\int_{t_{s}}^{t_{e}}\tau \left(t\right)dt}$$ (10a)

where τ(t) is the departure rate defined in formula (4), serving as a probability weight that reflects the likelihood of a departure occurring at time t. The average riding fee at node i is then:


$${\overset{―}{f}}_{i}=\sum_{j=1}^{n}P_{ij}\cdot {\overset{―}{f}}_{ij}$$ (10b)

where $\left[t_{s},t_{e}\right]$ is the operational time window.

The expected revenue at node i is the sum of expected riding fees generated by the bike-sharing deployed at that node:


$$I_{i}=min\left(q_{i},D_{i}\left({\overset{―}{f}}_{i}\right)\right)\cdot \sum_{j=1}^{n}P_{ij}\cdot {\overset{―}{f}}_{ij}$$ (11)

where $q_{i}$ is the number of bike-sharing deployed at node i, and $min\left(q_{i},D_{i}\left({\overset{―}{f}}_{i}\right)\right)$ reflects that the actual served demand cannot exceed either the supply or the adjusted demand.

When the number of bike-sharing deployed deviates from the optimal deployment number $q_{i}^{*}$, opportunity costs arise. Under-supply leads to lost revenue from unmet demand, while over-supply leads to idle resource costs. Since the marginal cost of unmet demand (lost customers, reduced service quality) is typically higher than that of idle resources, we adopt an asymmetric penalty:


$$V_{i}\left(q_{i}\right)=\left\{ \begin{array}{c} \alpha \cdot (q_{i}^{*}-q_{i}),\;q_{i}<q_{i}^{*}\\ \beta \cdot (q_{i}-q_{i}^{*},\;q_{i}\geq q_{i}^{*} \end{array}\right.$$ (12)

where α>β>0, reflecting the higher penalty for under-supply compared to over-supply. The optimal deployment number $q_{i}$ is determined by the adjusted demand: $q_{i}^{*}=\left\lceil D_{i}\left({\overset{―}{f}}_{i}\right)\right\rceil$. The opportunity cost function $V_{i}$ also serves as an implicit fairness regularization term: by penalizing deviations from the target allocation $q_{i}^{*}$ in both directions, the model discourages resource concentration at high-revenue stations and ensures a service level commensurate with local demand across all stations.

### 2.3. Joint optimization model for allocation and rebalancing

To overcome the limitation of independently optimizing allocation and rebalancing (which cannot guarantee global optimality), we propose a joint optimization model that integrates dynamic pricing revenue, opportunity cost, and dispatch cost into a unified objective function.

Overall Objective Function:


$$max\;\Pi =\sum_{i=1}^{n}I_{i}-\sum_{i=1}^{n}V_{i}\left(q_{i}\right)-Z$$ (13)

where Π is the total net profit, $\sum I_{i}$ is the total expected revenue, $\sum V_{i}$ is the total opportunity cost, and Z is the total rebalancing cost.

The total rebalancing cost is defined as:


$$Z=\sum_{\left(i,j\right)\in A}\sum_{k\in K}d_{ij}\cdot u\cdot x_{ij}^{k}+F\cdot \sum_{k\in K}\delta_{k}$$ (14)

where $\delta_{k}\in \left\{0,1\right\}$ is a binary decision variable indicating whether dispatch truck k is deployed ($\delta_{k}=1$) or not ($\delta_{k}=0$).

Allocation Constraints


$$q_{i}^{min}\leq q_{i}\leq q_{i}^{max},\;\forall i\in \{1,2,...,n\}$$ (15)

$$\sum_{i=1}^{n}q_{i}=m$$ (16)

$$\sum_{j=1}^{n}P_{ij}=1,\;\forall i\in \{1,2,...,n\}$$ (17)

where constraint (15) specifies the lower and upper bounds for bike-sharing deployment at each node (the lower bound $q_{i}^{min}$ ensures a minimum service level); constraint (16) ensures the total deployment equals the available fleet size m; constraints (17) is the row normalization conditions of the transition probability matrix, respectively.

Rebalancing Constraints


$$\sum_{k\in K}y_{i}^{k}\leq O_{i},\;\forall i\in I$$ (18)

$$S_{j}=O_{j}-\sum_{k\in K}y_{j}^{k},\;\forall j\in J$$ (19)

$$q_{j}^{min}\leq S_{j}\leq q_{j},\;\forall j\in J$$ (20)

$$y_{\text{0}}^{k}=Q_{\text{0}}^{k},\;\forall k\in K$$ (21)

$$\sum_{i\in I}y_{i}^{k}+\sum_{j\in J}y_{j}^{k}=0,\;\forall k\in K$$ (22)

$$\sum_{\left(i,j\right)\in A}d_{ij}\cdot x_{ij}^{k}\leq D,\;\forall k\in K$$ (23)

$$0\leq Q_{\text{0}}^{k}\leq V,\;\forall k\in K$$ (24)

$$Q_{i}^{k}+y_{j}^{k}-M(1-x_{ij}^{k})\leq Q_{j}^{k},\;\forall \left(i,j\right)\in A,\forall k\in K$$ (25a)

$$Q_{j}^{k}\leq Q_{i}^{k}+y_{j}^{k}+M(1-x_{ij}^{k}),\;\forall \left(i,j\right)\in A,\;\forall k\in K$$ (25b)

$$0\leq Q_{i}^{k}\leq V,\;\forall k\in K,\;\forall i\in I⋃J⋃0$$ (26)

$$\sum_{j\in I⋃J}x_{0j}^{k}\leq 1,\;\forall k\in K$$ (27)

$$\sum_{i\in I⋃J}x_{i0}^{k}\leq 1,\;\forall k\in K$$ (28)

$$x_{ij}^{k}\in \left\{0,1\right\},\;\forall \left(i,j\right)\in A,\forall k\in K$$ (29)

$$x_{ij}^{k}\leq \delta_{k},\;\forall \left(i,j\right)\in A,\;\forall k\in K$$ (30)

$$\delta_{k}\in \left\{0,1\right\},\;\forall k\in K$$ (31)

In the above rebalancing constraints, constraint (18) ensures that the number of bike-sharing picked up by all trucks at node i does not exceed the available quantity before dispatch, while constraint (19) defines the post-dispatch quantity at node j as the original quantity minus the net pickup/delivery by all trucks. Constraint (20) further requires that the post-dispatch quantity at each node falls between the minimum service level and the target allocation determined by the upper-level optimization. Constraints (21) and (24) together govern the truck’s loading condition: the former specifies the initial load when leaving the dispatch center, and the latter limits the load capacity to the maximum V. Constraint (22) enforces flow balance by requiring that, for each truck k, the total bikes loaded at pickup nodes and the total bikes unloaded at delivery nodes sum to zero. This is consistent with the sign convention that $y_{i}^{k}\geq 0$ at pickup nodes i∈I (truck gains bikes) and $y_{j}^{k}\leq 0$ at delivery nodes j∈J (truck loses bikes), ensuring no bikes are created or lost in transit, and Constraints (25a) and (25b) together enforce exact load tracking between consecutive nodes: when truck k travels from node i to node j ($x_{ij}^{k}=1$), the load upon leaving j equals the load upon leaving i adjusted by the loading/unloading amount $y_{j}^{k}$ at j; when $x_{ij}^{k}=0$, the big-M terms deactivate both constraints. Constraint (23) stipulates that the total driving distance of each truck cannot exceed the maximum threshold D, and Constraint (26) ensures the truck load remains within the feasible range [0,V] at all nodes throughout the route. The feasible range of $y_{i}^{k}$ is implicitly determined by constraints (25a), (25b), and (26) jointly. Constraints (27) and (28) ensure that each dispatch truck departs from and returns to the dispatch center, and constraint (29) declares $x_{ij}^{k}$ as a binary decision variable. Constraint (30) ensures that if truck k is not deployed, it cannot traverse any arc. Constraint (31) declares $\delta_{k}$ as a binary variable.

Since the joint optimization model involves both continuous variables (pricing, allocation) and integer variables (routing), and the price-demand feedback creates nonlinearity, we adopt an iterative decomposition approach:

Step 1 (Pricing and Allocation): Given an initial allocation $q_{i}$, compute the dynamic pricing fees $f_{ij}\left(t\right)$ using formula (8), the adjusted demand $D_{ij}\left({\overset{―}{f}}_{i}\right)$ using formula (9), and the expected revenue $I_{i}$ using formula (11). Optimize the allocation $q_{i}$ to maximize $\sum I_{i}-\sum V_{i}$.

Step 2 (Rebalancing): Given the target allocation $q_{i}$ from Step 1 and the current distribution $O_{i}$, solve the vehicle routing problem to minimize the dispatch cost Z subject to constraints (18)-(31). The allocation $q_{i}$ determined in Stage 1 directly defines the rebalancing targets $need_{i}=q_{i}-O_{i}$ in Stage 2, creating an endogenous coupling in which pricing decisions and routing operations are jointly optimized through the unified objective function (13).

Step 3 (Joint Evaluation): Compute the total net profit Π from formula (13). If the profit improves compared to the previous iteration, update the solution; otherwise, adjust the pricing variance $\sigma_{t}$ and return to Step 1.

Step 4 (Convergence): Repeat Steps 1–3 until the change in Π between consecutive iterations falls below a predefined threshold δ, or the maximum number of iterations is reached.

This iterative approach ensures that the allocation considers the rebalancing cost, and the pricing responds to the supply-demand dynamics, achieving an approximate joint optimum.

## 3. Algorithm design

### 3.1. Dynamic pricing algorithm

Based on the dynamic pricing model proposed in Section 2.2, we design a dynamic pricing algorithm that computes the riding fee, adjusts demand through price elasticity, and evaluates the expected revenue for each node. The algorithm proceeds through the following stages.

First, the predicted demand values of all nodes are sorted in ascending order to determine the importance ranking of each node. Let $w_{i}$ denote the ranking of node i. For each pair of nodes i and j, the asymmetric directional weight is computed as $w_{ij}=w_{j}/max\left(w_{i},w_{j}\right)$, which ensures that rides toward high-demand nodes receive higher weights.

Second, the transition probability between nodes is estimated from historical OD data. For each origin node i, the transition probability to destination node j is calculated as $P_{ij}=N_{ij}/\sum_{j=1}^{n}N_{ij}$, where $N_{ij}$ is the observed number of trips from node i to node j. If historical OD data is insufficient, the independence approximation $P_{ij}\approx b_{i}\cdot r_{j}$ can be used as a fallback.

Third, the normalized departure time pricing weight is generated for each bike-sharing. For the k -th bike-sharing departing at time $t_{k}$, the pricing weight is:


$$\psi \left(t_{k}\right)=1+\alpha \cdot e^{-\frac{(t_{k}-\mu_{t}{)}^{\text{2}}}{2\sigma_{t}^{\text{2}}}}$$

where $\mu_{t}$ and $\sigma_{t}$ are the mean and standard deviation obtained from historical data fitting, and α>0 is the peak pricing premium coefficient. This weight ranges within [1,1+α], reaching its maximum 1+α at peak time $t_{k}=\mu_{t}$ and approaching the standard rate 1 during off-peak periods. The riding fee for a user departing at time $t_{k}$ from node i to node j is then $f_{ij}\left(t_{k}\right)=w_{ij}\cdot d_{ij}\cdot \psi \left(t_{k}\right)\cdot c_{\text{0}}$. The time-averaged fee from node i to node j is approximated as:


$${\overset{―}{f}}_{ij}\approx \frac{\text{1}}{q_{i}}\sum_{k=1}^{q_{i}}f_{ij}\left(t_{k}\right)=w_{ij}\cdot d_{ij}\cdot c_{\text{0}}\cdot \frac{\text{1}}{q_{i}}\sum_{k=1}^{q_{i}}\psi \left(t_{k}\right)$$

Fourth, the price elasticity mechanism is applied to adjust the demand at each node. The adjusted demand is $D_{i}^{adj}=D_{i}^{\text{0}}\cdot ({\overset{―}{f}}_{i}/f_{\text{0}}{)}^{-\varepsilon }$, where $D_{i}^{\text{0}}$ is the baseline demand, $f_{\text{0}}$ is the reference fee, and ε is the price elasticity coefficient. The expected revenue at node i is then $I_{i}=min\left(q_{i},D_{i}^{adj}\right)\cdot {\overset{―}{f}}_{i}$, reflecting that the actual served demand cannot exceed either the supply or the adjusted demand. The optimal deployment number at node i is $q_{i}^{*}=\left\lceil D_{i}^{adj}\right\rceil$, where ⌈⬝⌉ denotes the ceiling function, ensuring $q_{i}^{*}$ is an integer consistent with the discrete nature of bike-sharing deployment. The asymmetric opportunity cost is computed as $V_{i}=\alpha \cdot (q_{i}^{*}-q_{i})$ when $q_{i}<q_{i}^{*}$, or $V_{i}=\beta \cdot (q_{i}-q_{i}^{*})$ when $q_{i}\geq q_{i}^{*}$, where α>β>0. Finally, the total allocation profit is evaluated as $\Pi_{alloc}=\sum_{i=1}^{n}I_{i}-\sum_{i=1}^{n}V_{i}$.

### 3.2. Iterative joint optimization based on PSO-LNS

To solve the joint optimization model proposed in Section 3.3, this paper designs an iterative framework that alternates between the allocation optimization and the rebalancing optimization. The rebalancing sub-problem is solved using an improved Particle Swarm Optimization (PSO) algorithm based on Large-scale Neighborhood Search (LNS), significantly influenced by destroy-and-repair metaheuristic algorithms [31–33].

The overall iterative process is as follows. The algorithm initializes the best profit $\Pi_{best}=-\infty$ and the iteration counter. In each outer iteration, the dynamic pricing algorithm described in Section 4.1 is first executed with the current pricing variance $\sigma_{t}$ to obtain the allocation scheme $q_{i}$, the expected revenue $I_{i}$, the opportunity cost $V_{i}$, and the allocation profit $\Pi_{alloc}=\sum I_{i}-\sum V_{i}$.

Next, the rebalancing sub-problem is solved using the PSO-LNS algorithm. A set of particles is initialized, where each particle encodes a dispatch plan including node-to-truck assignments and visiting sequences. The particle positions and velocities are initialized randomly within feasible bounds. For each particle, the fitness is evaluated using the joint objective $\Pi =\Pi_{alloc}-Z$, where Z is the rebalancing cost of the dispatch plan encoded by the particle. In each generation, the velocity of each particle is updated according to the standard PSO formula: $V_{d}^{k+1}=\omega \cdot V_{d}^{k}+c_{\text{1}}r_{\text{1}}(P_{id}-X^{k})+c_{\text{2}}r_{\text{2}}(P_{gd}-X^{k})$, where ω is the inertia weight, $c_{\text{1}}$ and $c_{\text{2}}$ are the learning factors, $r_{\text{1}}$ and $r_{\text{2}}$ are random numbers in [0,1], $P_{id}$ is the personal best position, and $P_{gd}$ is the global best position. The particle position is then updated as $X^{k+1}=X^{k}+V^{k+1}$. If the new fitness exceeds the personal or global best, the corresponding records are updated. This inner loop repeats until the maximum number of generations is reached.

After the PSO inner loop converges, a relocate local search operation is performed on the global best particle to further improve the solution quality. This operation first identifies nodes with constraint violations by computing the loss value at each node: for under-supplied nodes where $q_{i}<q_{i}^{*}$, the loss is $loss_{i}=\alpha \cdot (q_{i}^{*}-q_{i})$; for all other nodes, the loss is zero. The node with the largest loss value is selected as the target for improvement. A GENE operation is then performed: two routes are randomly selected from the current dispatch plan, and a node exchange is executed between them. The new dispatch plan is evaluated using the joint objective $\Pi_{new}=\sum I_{i}-\sum V_{i}-Z$. If $\Pi_{new}>\Pi_{old}$, the new route is accepted; otherwise, the original route is retained. This mechanism ensures that local search decisions are consistent with the overall profit maximization goal, and that under-supplied nodes are prioritized for rebalancing due to the higher penalty coefficient α>β.

After the rebalancing optimization and local search are completed, the total net profit is computed as $\Pi =\Pi_{alloc}-Z_{best}$. If $\Pi >\Pi_{best}$, the best solution is updated with the current allocation scheme and dispatch routes. Otherwise, the pricing variance $\sigma_{t}$ is perturbed by $\sigma_{t}=\sigma_{t}\cdot \left(1+\eta \cdot random\left(-1,1\right)\right)$, where η is the perturbation factor, to explore alternative pricing strategies in the next iteration. The outer loop terminates when the change in profit between consecutive iterations falls below a predefined threshold δ, or when the maximum number of outer iterations is reached. This iterative approach ensures that the allocation considers the rebalancing cost, and the pricing responds to the supply-demand dynamics, achieving an approximate joint optimum. The outer loop terminates when the improvement in net profit between consecutive iterations falls below δ=0.5, or when the maximum of 20 iterations is reached. The PSO inner loop runs for a maximum of 200 generations per outer iteration. The hyperparameters are summarized in Table 2.

**Table 2:** Hyperparameter Settings of the Proposed PSO-LNS Algorithm.

Parameter	Description	Value
N	Swarm size	50
$G_{max}$	Maximum PSO generations	200
$w_{\text{0}}$	Initial inertia weight	0.9
$w_{damp}$	Inertia damping factor	0.99
$c_{\text{1}}$	Cognitive coefficient	1.5
$c_{\text{2}}$	Social coefficient	2.0
$T_{LNS}$	Number of LNS iterations	50
ρ	Removal fraction	15%
$D_{rand}$	Randomization degree	6
δ	Convergence threshold	0.5
$out_{max}$	Maximum outer iterations	20

The complete source code is available at https://github.com/John-von/Optimization-problem-of-bike-sharing-system-rebalancing-based-on-dynamic-pricing.git.

## 4. Numerical studies

**Fig 1:**
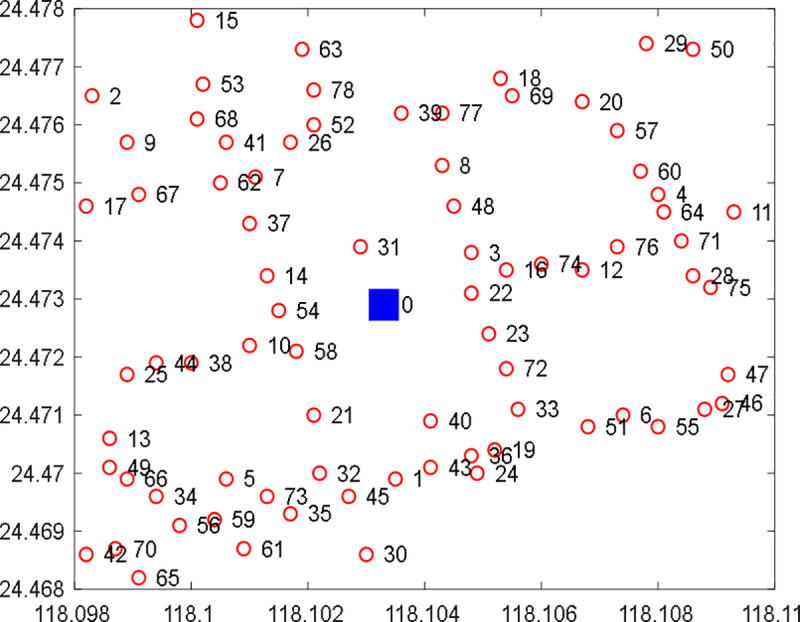
Distribution of bike-sharing stations.

The analysis sample is based on shared bicycle order information in Xiamen City from 6:00–10:00 on December 21, 2020, within the latitude range [24.468, 24.478] and longitude range [118.098, 118.11]. The spatial coverage of the dataset, while geographically compact, encompasses a representative high-density commuting corridor with sufficient station density and demand heterogeneity for model validation purposes. The figure includes one shared bicycle dispatch center, labeled as 0, represented by a blue square; 78 shared bicycle nodes, labeled 1–78, represented by red dots, as shown in Fig 1. For detailed order data analysis and data processing, please refer to [30]. Although the data was collected during the COVID-19 pandemic, China had largely resumed normal urban commuting by December 2020, and the morning peak commuter demand exhibits structural stability less affected by pandemic-era behavioral shifts. More critically, the validity of the DSDRO framework depends on the existence of spatiotemporal demand heterogeneity—a feature that persists across demand regimes—rather than on any specific absolute demand level. It should be noted that the findings from this case study demonstrate the methodological feasibility of DSDRO, not universally applicable conclusions. Validation on additional datasets remains important future work.

To solve the dispatch plan for 1283 bike-sharing across 78 nodes, we assumed a maximum of 10 dispatch trucks at the dispatch center, with each truck having a maximum loading capacity of 100 bike-sharing, a maximum travel distance of 200 km, an average travel speed of 30 km/h, an operational cost of 100 USD per use for managing a dispatch truck, and a unit distance dispatch cost of 1 USD/km. The base parameters of the algorithm were chosen as the most reasonable values through repeated analysis of the results.

**Fig 2:**
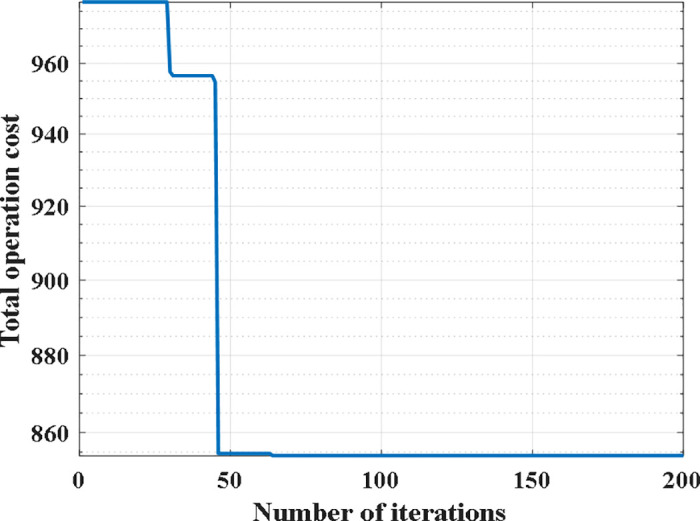
Genetic algorithm optimization process.

**Fig 3:**
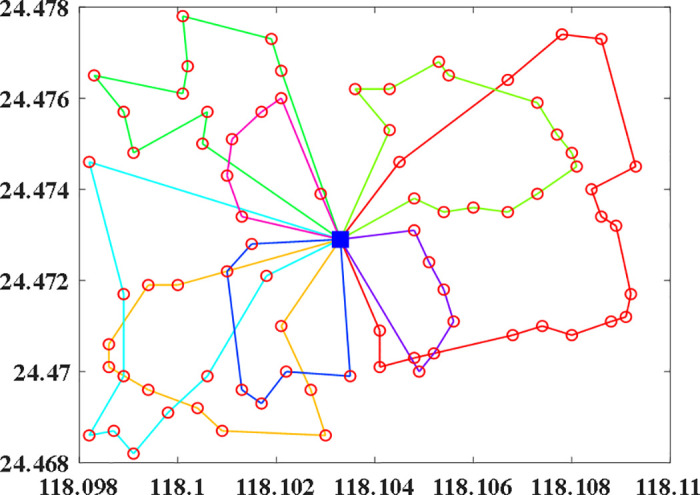
Dispatching vehicle route.

To verify the feasibility of the model and algorithm in this paper, first, based on the prediction of historical order data, a genetic algorithm was used to solve the traditional dispatch problem with the objective of minimizing total dispatch cost. The model converged after 61 iterations as shown in Fig 2, resulting in the optimal dispatch route of the dispatch trucks for the 78 sites as shown in Fig 3. The specific route is shown in Table 3.

**Table 3:** Optimal rebalancing scheme.

Rebalancing Scheme	Optimal Solution
**Route 1**	0- > 40- > 43- > 36- > 51- > 6- > 55- > 27- > 46- > 47- > 75- > 28- > 71- > 11- > 50- > 29- > 20- > 48- > 0
**Route 2**	0- > 38- > 44- > 13- > 49- > 34- > 59- > 61- > 30- > 45- > 21- > 0
**Route 3**	0- > 8- > 39- > 77- > 18- > 69- > 57- > 60- > 4- > 64- > 76- > 12- > 74- > 16- > 3- > 0
**Route 4**	0- > 62- > 41- > 67- > 9- > 2- > 68- > 53- > 15- > 63- > 78- > 0
**Route 5**	0- > 58- > 5- > 56- > 65- > 70- > 42- > 66- > 25- > 17- > 0
**Route 6**	0- > 54- > 10- > 73- > 35- > 32- > 1- > 0
**Route 7**	0- > 22- > 23- > 72- > 33- > 19- > 24- > 0
**Route 8**	0- > 14- > 37- > 7- > 26- > 52- > 31- > 0

**Fig 4:**
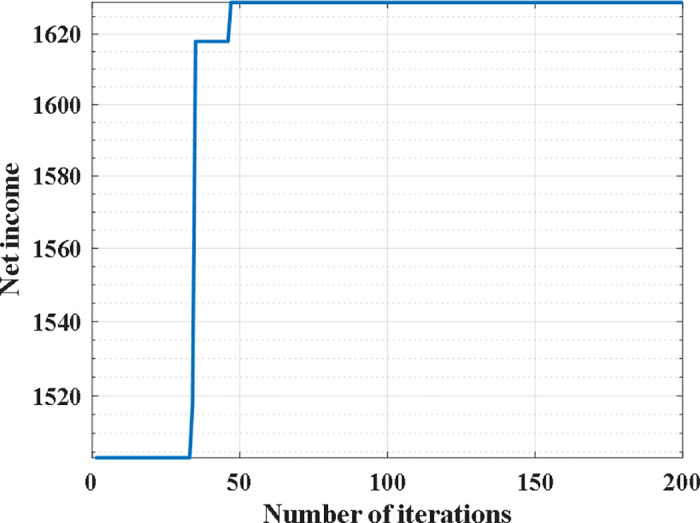
Particle swarm optimization process.

**Fig 5:**
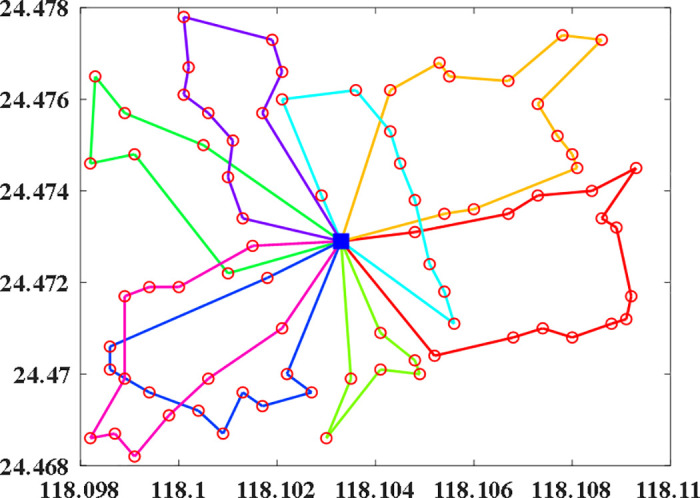
Dispatching vehicle route.

Since the importance of each bike-sharing node varies, the demand for bike-sharing at different nodes will also differ, and the riding transition probabilities of bike-sharing in different areas will affect the revenue generated by the number of bike-sharing deployed. Therefore, we tested the above example using the model and algorithm proposed in this paper. The test results are shown in Fig 4, the specific route is shown in Table 4. As can be seen from the figure, the algorithm achieved good convergence at the 50th generation and produced the optimal dispatch plan for the 78 nodes, as shown in Fig 5. This demonstrates that the model and algorithm proposed in this paper can accurately consider the demand of different nodes and the transition probabilities of different areas, providing enterprises with more precise and efficient dispatch plans.

**Table 4:** Optimal rebalancing scheme.

Rebalancing Scheme	Optimal Solution
**Route 1**	0- > 22- > 12- > 76- > 71- > 11- > 28- > 75- > 47- > 46- > 27- > 55- > 6- > 51- > 19- > 0
**Route 2**	0- > 77- > 18- > 69- > 20- > 29- > 50- > 57- > 60- > 4- > 64- > 74- > 16- > 0
**Route 3**	0- > 1- > 30- > 43- > 24- > 36- > 40- > 0
**Route 4**	0- > 10- > 67- > 17- > 2- > 9- > 62- > 0
**Route 5**	0- > 31- > 52- > 39- > 8- > 48- > 3- > 23- > 72- > 33- > 0
**Route 6**	0- > 32- > 45- > 35- > 73- > 61- > 59- > 34- > 49- > 13- > 58- > 0
**Route 7**	0- > 26- > 78- > 63- > 15- > 53- > 68- > 41- > 7- > 37- > 14- > 0
**Route 8**	0- > 21- > 5- > 56- > 65- > 70- > 42- > 66- > 25- > 44- > 38- > 54- > 0

**Fig 6:**
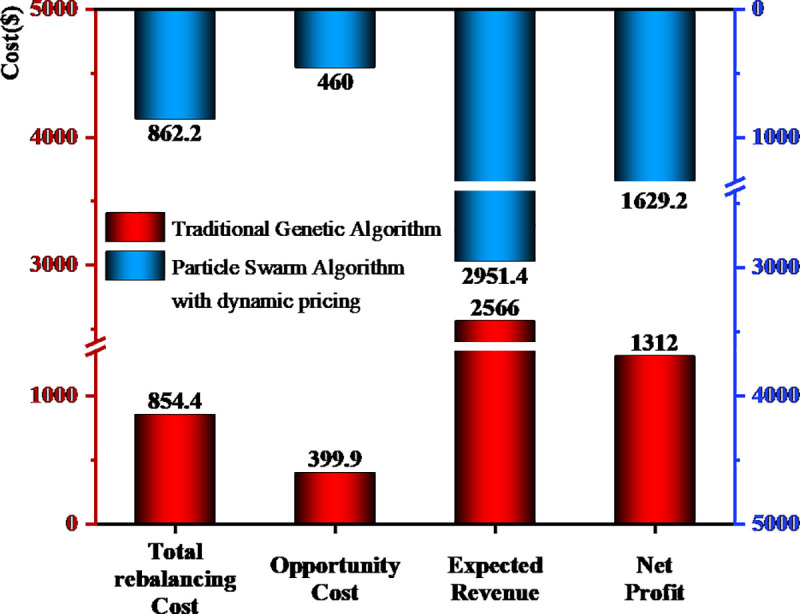
Comparison chart of key indicators.

Fig 6 shows a comparison of key indicators, where it can be seen that the improved particle swarm algorithm exhibits significant advantages over the traditional genetic algorithm in core performance metrics. Although total travel distance increased from 54.4 km to 62.2 km and rebalancing costs rose from 854.4 to 862.2, expected revenue improved to 2,951.4, a gain of 15.51%, with net profit rising from 1,312–1,629.2, an increase of 24.18%. This outcome is consistent with the design logic of the proposed framework: the demand-elastic allocation concentrates bikes at high-demand nodes, reducing spatial mismatches and increasing ridership revenue, while the PSO-LNS optimizer consolidates rebalancing routes to keep additional costs minimal.

**Fig 7:**
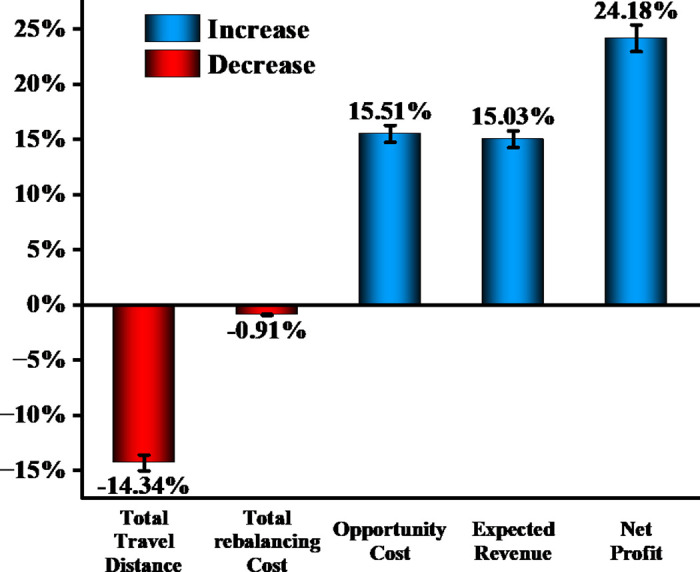
Optimization improvement rate chart.

Fig 7 illustrates the percentage change in each key metric relative to the genetic algorithm baseline, revealing an asymmetric pattern in which rebalancing cost increases by only 0.91% while expected revenue and net profit improve by 15.51% and 24.18%, respectively. It is worth noting that the net profit growth rate exceeds the revenue growth rate, which is attributable to the operating leverage effect inherent in the cost structure. While revenue increases by 385.4, total costs increase by only 67.9, since the rebalancing cost—which contains a substantial fixed vehicle dispatch component—grows by merely 0.91%. This demonstrates that the dynamic pricing mechanism not only increases ridership revenue but also improves the overall cost efficiency of the rebalancing operation, thereby amplifying profit margins beyond the level of revenue growth alone.

**Fig 8:**
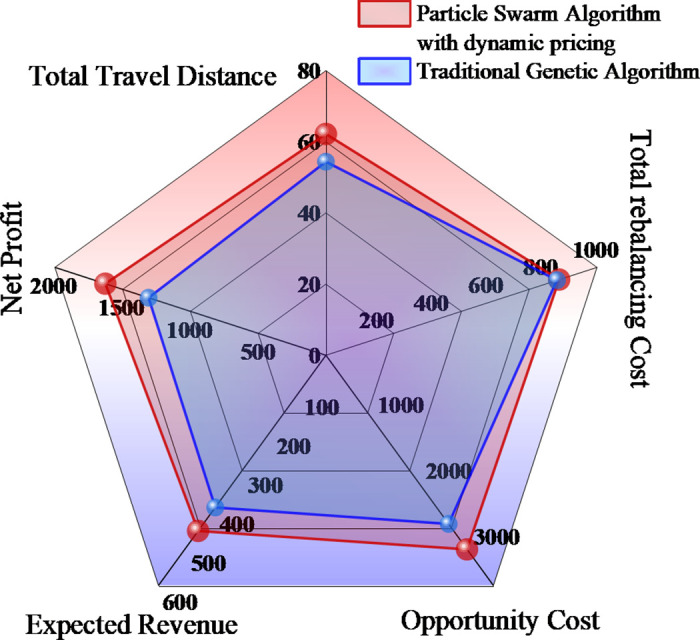
Comprehensive performance radar chart.

Fig 8 evaluates performance differences between the two algorithms across five dimensions, with the improved algorithm achieving improvements across all dimensions, with revenue and profit gains of 15.51% and 24.18% respectively. The geometric configuration of the radar chart shows that the improved algorithm forms a larger and more balanced pentagon, indicating coordinated development across all performance dimensions. Particularly notable improvements are observed in revenue level and profit performance dimensions, while cost efficiency, resource utilization, and overall effectiveness dimensions also demonstrate stable performance enhancements. This comprehensive performance improvement fully demonstrates the synergistic effects of dynamic pricing strategies and optimized scheduling algorithms, validating the superior performance and well-balanced developmental characteristics of the improved algorithm in multi-objective optimization problems.

**Fig 9:**
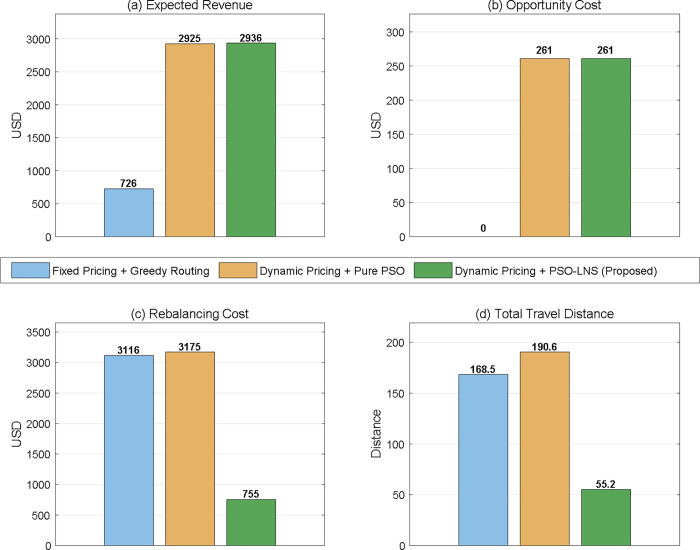
Performance Comparison of Three Methods.

To validate the contribution of each component in the proposed framework, three comparative experiments were conducted(as Fig 9): fixed pricing with greedy routing (Method 1), dynamic pricing with pure PSO (Method 2), and the proposed dynamic pricing with PSO-LNS (Method 3). The results demonstrate that, compared with Method 1, the introduction of the dynamic pricing mechanism increases expected revenue by over 300%, confirming its effectiveness in capturing spatiotemporal demand heterogeneity. Compared with Method 2, the incorporation of the LNS component reduces rebalancing cost by 76.21% and shortens total travel distance by 71.0%, demonstrating the superior deep-search capability of large neighborhood search in route optimization. These results highlight a significant synergistic effect between the dynamic pricing module and the PSO-LNS algorithm, as neither component alone is sufficient to achieve optimal system performance, thereby further substantiating the necessity and superiority of the proposed two-stage joint optimization framework. To further validate the algorithmic reliability, we conducted 10 independent runs of the proposed PSO-LNS with different random seeds(The ten runs use the following random seeds: 42, 7, 13, 21, 55, 88, 101, 137, 200, and 256, which are fixed prior to each run to ensure reproducibility). Table 5 reports the mean, standard deviation, and coefficient of variation (CV) for all key metrics. All CV values remain below 5%, confirming that the algorithm produces consistent and reproducible results across independent runs.

**Table 5:** Statistical Summary of 10 Independent Runs.

Metric	Mean	Std Dev	CV (%)
Expected Revenue	2933.21	14.78	0.50
Opportunity Cost	261.00	0.00	0.00
Rebalancing Cost	766.25	32.98	4.30
Net Profit	1905.96	38.23	2.01
Total Travel Distance	56.25	1.97	3.50
Trucks Used	7.10	0.32	4.45

**Fig 10:**
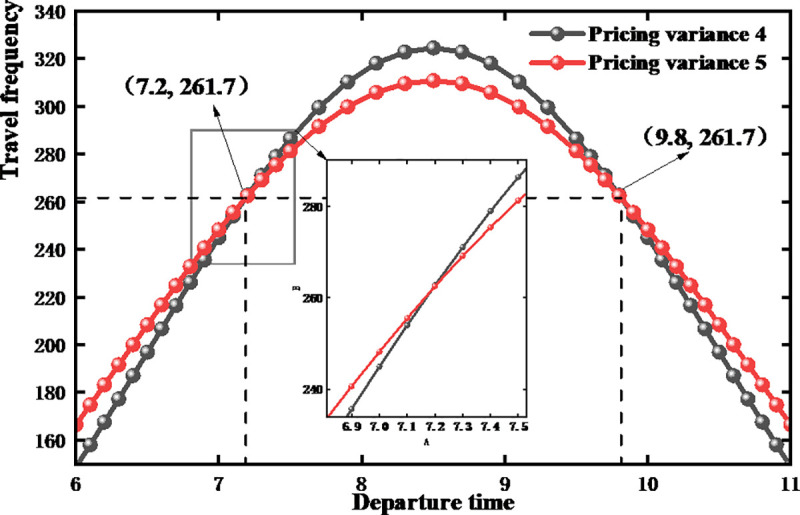
Impact of different pricing variance on the use of bike-sharing.

Fig 10 shows the sensitivity of bike-sharing users to different charges over time, representing the frequency of bike-sharing use during different periods under various pricing scenarios. By dynamically adjusting pricing—that is, by changing the variance in the normal distribution of pricing, it is possible to effectively differentiate users’ demand stickiness for bike-sharing. As seen in the figure, the area enclosed by the curves represents the cumulative usage of bike-sharing, with both curves encompassing an area corresponding to the regional deployment number of 1,283 bike-sharing. In the [6, 7.2] time period, adjusting the pricing variance can cause approximately 1.8% of travelers to travel earlier or later, thereby reducing the number of people traveling during peak periods. This indicates that a smaller variance in charges leads most users to prefer clustered departures, which can easily result in larger peaks during rush hours. This indirectly shows that under low pricing standards, the differentiation in demand stickiness for bike-sharing among users is small, leading to inefficient resource utilization. Conversely, a larger variance can disperse the rate of departures, further differentiating the stickiness of user demand for bike-sharing. The reason for this outcome lies in the differing sensitivities of users to pricing standards. Considering the urgency of their needs, some users may have a higher tolerance for pricing, while others, due to non-urgent needs and pricing sensitivity, may avoid using bike-sharing during higher pricing periods.

**Fig 11:**
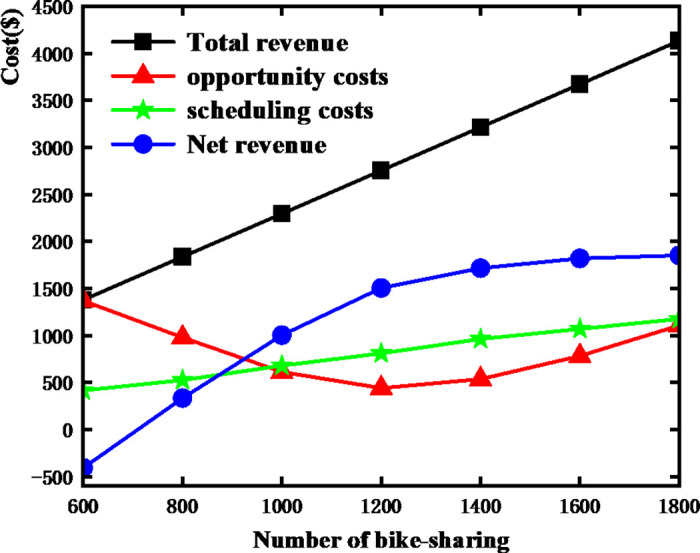
Trend of revenue and cost changes in the bike-sharing system under different deployment quantities.

Under the condition of keeping other parameters constant, we study the impact of different levels of bike-sharing deployment on the system’s cost and revenue. The specific trend of changes is shown in Fig 11. As the number of bike-sharing increases, profit and revenue initially increase and then decrease, while the overall transportation cost remains relatively unchanged. When the number of deployed bike-sharing is only 60% of the demand level, the enterprise operates at a loss in the area, meaning that the operational costs exceed the revenue generated by the bike-sharing in the area. This implies that there are fewer idle bike-sharing, resulting in higher opportunity costs and costs associated with unmet demand in the area. Therefore, the enterprise does not need to specifically dispatch bike-sharing in this area and can wait for users to self-dispatch, or dispatch only when more bike-sharing accumulate as idle. When the number of bike-sharing reaches 125% of the example level, the net profit begins to decline, indicating an excess of idle bike-sharing. Even if the enterprise relocates these idle bike-sharing to areas with higher demand, the excessive number of bike-sharing will still lead to a decrease in profit. In this case, the enterprise should consider relocating the surplus bike-sharing to other areas, or even reflect on whether too many bike-sharing have been deployed, leading to market saturation.

**Fig 12:**
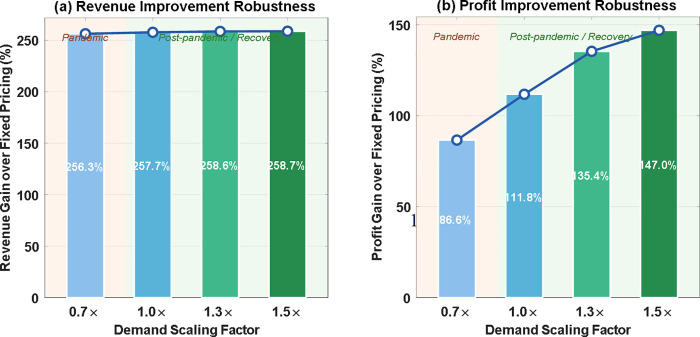
DSDRO vs. Fixed Pricing under Different Demand Regimes.

Fig 12 presents the robustness of the DSDRO framework across four demand scaling scenarios, measured by the revenue and profit gains relative to the fixed pricing baseline. Two notable patterns emerge. First, the revenue gain remains remarkably stable across all demand regimes, ranging from 256.30% to 258.75%—a variation of only 2.45 percentage points—indicating that the superiority of the dynamic pricing mechanism is independent of the absolute demand level. Second, the profit gain increases monotonically with demand, suggesting that the proposed framework becomes increasingly advantageous under demand recovery and post-pandemic high-demand conditions. These results demonstrate that the proposed framework maintains stable—and progressively stronger—performance advantages across a wide range of demand conditions, confirming its adaptability to varying operational environments.

## 5. Conclusion

In this study, we proposed a two-stage joint optimization model for bike-sharing implementing dynamic pricing, considering user demand stickiness and enterprise revenue maximization. The key decisions in this problem are the charging for bike-sharing at different times and nodes, and the initial dispatch allocation of bike-sharing. We introduced a Dynamic pricing-based Stochastic Demand-Response Optimization (DSDRO) model, aiming to effectively price for different user demand stickiness while optimizing the utilization of bike-sharing resources.

Specifically, we first analyzed the dynamic evolution of bike-sharing in time and space based on order data, quantifying the importance of each node. Next, we established a dynamic pricing model for bike-sharing’s stochastic distribution, fully considering factors such as departure time and node importance, to balance and quantify users’ riding costs. Finally, we proposed a two-stage joint optimization model and designed an improved Particle Swarm Optimization algorithm based on Large-scale Neighborhood Search (PSO-LNS) to solve the model. We tested the performance of the DSDRO model through comprehensive numerical experiments with bike-sharing order data provided by the 2021 Digital China Innovation Contest. Compared to the traditional genetic algorithm baseline, the DSDRO model increases expected revenue by 15.51% and net profit by 24.18% under identical resource conditions. An ablation study further demonstrates that dynamic pricing alone increases expected revenue by over 300% relative to fixed pricing, while the LNS component reduces rebalancing cost by 76.21% relative to pure PSO, confirming the necessity and synergistic effect of the two-stage joint optimization framework. Algorithm stability was verified through 10 independent runs, with all performance metrics exhibiting a coefficient of variation below 5%. Robustness analysis across four demand scaling scenarios shows that the revenue gain remains stable at 256%–259%, suggesting that the proposed framework maintains its performance advantages across the tested range of demand conditions. Additionally, sensitivity analysis on deployment levels reveals that when deployment falls below 60% of demand, the enterprise operates at a loss; when deployment exceeds 125% of demand, net profit begins to decline due to resource over-supply.

Our study also has some limitations. Firstly, the model rests on several simplifying assumptions, including a single dispatch center, no weather or traffic disruptions, no bike damage or recovery, and one visit per truck per node. These assumptions are standard in initial modeling studies but reduce real-world complexity; the findings should therefore be viewed as promising under simplified conditions rather than as a full real-world deployment test. Secondly, we only considered the dynamic pricing and dispatch allocation problems of BSS from the perspective of enterprise survival, lacking consideration of user acceptance, which will be further addressed in our next work. Thirdly, we used PSO-LNS to solve the problem considering the actual scale of the example; in the future, other gradient-based algorithms could be considered for solving larger-scale instances. Fourthly, the empirical validation is based on a single city (Xiamen), a single date, and a single morning peak window. While the robustness analysis across demand scaling factors provides partial evidence of generalizability, the reported performance gains should be interpreted as indicative of the method’s potential rather than as evidence of broad practical effectiveness. Future work should validate the proposed approach on datasets from multiple cities and time periods.

## Supporting information

S1 File Dataset and MATLAB implementation of the DSDRO algorithm. This file contains the station coordinates, station demand data, and the source code for the proposed algorithm, including the PSO-LNS solver, ablation study scripts, and 10-run statistical validation scripts. The same materials are also publicly available at: https://github.com/John-von/Optimization-problem-of-bike-sharing-system-rebalancing-based-on-dynamic-pricing.git. The contents of this file and the GitHub repository are identical.
(ZIP)
